# New and Emerging Biologics and Jak Inhibitors for the Treatment of Prurigo Nodularis: A Narrative Review

**DOI:** 10.3390/medicina61040631

**Published:** 2025-03-29

**Authors:** Matteo Bianco, Francesco D’Oria, Costanza Falcidia, Giulio Foggi, Elena Matteodo, Sara Di Giulio, Paola Facheris, Luciano Ibba, Chiara Perugini, Mario Valenti, Carlo Alberto Vignoli, Antonio Costanzo, Alessandra Narcisi, Luigi Gargiulo

**Affiliations:** 1 Dermatology Unit, IRCCS Humanitas Research Hospital, 20089 Rozzano, Italy; matteo.bianco@humanitas.it (M.B.); francesco.doria@humanitas.it (F.D.); costanza.falcidia@humanitas.it (C.F.); giulio.foggi@humanitas.it (G.F.); elena.matteodo@humanitas.it (E.M.); sara.digiulio@humanitas.it (S.D.G.); paola.facheris@humanitas.it (P.F.); luciano.ibba@humanitas.it (L.I.); chiara.perugini@humanitas.it (C.P.); mario.valenti@hunimed.eu (M.V.); carlo.vignoli@humanitas.it (C.A.V.); antonio.costanzo@hunimed.eu (A.C.); alessandra.narcisi@humanitas.it (A.N.); 2 Department of Biomedical Sciences, Humanitas University, Pieve Emanuele, 20072 Milan, Italy

**Keywords:** prurigo nodularis, biologics, monoclonal antibodies, JAK inhibitors, neuroimmune dysregulation, targeted therapy

## Abstract

Prurigo nodularis (PN) is a chronic dermatological condition characterized by intensely pruritic nodules resulting from repeated scratching. Its pathogenesis involves neuroimmune dysregulation, inflammatory cytokines, and neural proliferation. Conventional treatments often provide limited relief, necessitating novel therapeutic approaches. This narrative review explores emerging biologics and small molecules for PN treatment, assessing their mechanisms, efficacy, and safety. A comprehensive literature search was conducted using PubMed, Google Scholar, and Web of Science for relevant studies up to February 2025. Additionally, ongoing clinical trials were identified through a verified international website. The search terms included “prurigo nodularis”, “biologic treatments”, “monoclonal antibodies”, “small molecules”, and “JAK inhibitors”. Among new treatment options, dupilumab, an IL-4 receptor antagonist, and nemolizumab, an IL-31 receptor inhibitor, demonstrated significant efficacy in reducing pruritus and lesion severity in PN patients. Other promising monoclonal antibodies include vixarelimab (OSMRβ inhibitor) and barzolvolimab (KIT inhibitor). Small molecules such as JAK inhibitors (upadacitinib, povorcitinib) also show potential by modulating inflammatory pathways. Clinical trials highlight their efficacy, safety, and long-term benefits. Emerging biologics and small molecules represent a transformative approach for PN management, offering targeted therapies that address underlying immunological and neurological mechanisms. Ongoing research and long-term studies are crucial to optimizing treatment strategies and improving patient outcomes.

## 1. Introduction

Prurigo nodularis (PN) is a chronic skin disorder characterized by persistent itchy nodules caused by repetitive scratching or picking of the skin over an extended period [1]. This condition typically appears in middle age or later and is diagnosed more frequently in females than in males [2]. The nodules are intensely itchy, likely due to hypertrophy and an increased density of substance P-positive nerves in the affected skin, perpetuating the itch–scratch cycle [3].

PN is linked to several dermatological conditions, such as atopic dermatitis (AD) and xerosis, along with systemic diseases like hyperthyroidism, hepatic or renal dysfunction, congestive heart failure, chronic hepatitis C, human immunodeficiency virus (HIV), and lymphoma [4,5]. Psychological factors, including emotional distress, obsessive–compulsive disorder, and depression, may also play a role in repetitive scratching, worsening the condition [6,7]. Furthermore, genetic predisposition and environmental triggers such as infections, vaccinations, and immunotherapies have been implicated in the pathogenesis of PN [8]. This multimorbidity may elevate the risk of developing PN, and conversely, PN may exacerbate the onset of additional comorbidities due to chronic systemic inflammation [9]. Despite these associations, the exact cause of PN remains uncertain, with various hypotheses proposed (Table 1).

The first genetic study in patients with PN identified a distinct polygenic risk score, indicating that a combination of genetic susceptibility and environmental factors contribute to disease development [10].

Recent studies have highlighted the role of immune dysregulation in PN, with inflammation driven by cytokines released from T-helper (Th)1, Th2, Th17, and Th22 cells [11]. The inflammatory infiltrate includes neutrophils, basophils, mast cells, eosinophils, and fibroblasts. Mast cells contribute to pruritus and neural proliferation by releasing inflammatory mediators and neurotrophins [12]. However, histamine is unlikely to be the primary driver of pruritus in PN, as the condition often exhibits poor responsiveness to antihistamines [13]. Eosinophils release neurotrophins, chemokines, and cytokines, while eosinophil-specific neurotoxins are elevated in PN lesions, potentially inducing nerve damage and contributing to pruritus [12].

Cytokines activate cutaneous nerve fibers, keratinocytes, macrophages, mast cells, and eosinophils, contributing to the pathogenesis of PN through the Janus kinase–signal transducer and activator of the transcription protein (JAK–STAT) pathway [14]. IL-4 and IL-13 have been shown to directly activate sensory neurons, increasing the sensation of itch, while IL-31 and oncostatin M receptor β (OSMRβ) play a crucial role in neuronal growth and inflammatory cell activation. Additionally, periostin, an extracellular matrix protein involved in type 2 inflammation, has been found in increased levels in PN lesions and correlates with pruritus severity [15]. Neurocutaneous involvement has also been proposed as a key component in PN pathogenesis. Earlier studies identified an increased number of nerve fibers in the papillary dermis, suggesting a neurocutaneous mechanism. Neural dysregulation is also evident in PN, with thickened and hyperplastic protein gene product 9.5-positive C fibers in the dermis, reduced intraepidermal nerve fiber density, and increased NGF, which contributes to heightened pruritus [16]. Recent insights into PN pathogenesis have also indicated potential involvement of the gut–skin axis. Emerging evidence suggests dysbiosis and altered microbiota composition might influence systemic inflammation and immune response, thus potentially exacerbating cutaneous symptoms in PN. Exploring these microbiome interactions could open new avenues for targeted therapeutic strategies [1]. The evaluation of PN typically relies on detailed patient history and thorough physical examination, focusing on the characteristic distribution and morphology of nodules [1,3]. Validated tools, such as the visual analog scale (VAS) for assessing itch intensity and dermatology-specific quality-of-life questionnaires, help quantify symptom severity and treatment efficacy. Additionally, histological examination through skin biopsy can aid differential diagnosis by revealing characteristic features, such as epidermal hyperplasia, hyperkeratosis, dermal fibrosis, and a dense inflammatory infiltrate [1,3].

## 2. Methods

The aim of this narrative review is to provide a comprehensive overview of recently approved and emerging treatments for PN, including both monoclonal antibodies and JAK inhibitors. English-language medical literature research was conducted by utilizing PubMed, Google Scholar, and Web of Science databases from the earliest records through February 2025. Also, we have conducted research on ongoing and novel clinical trials utilizing the website ClinicalTrials.gov. The search strategy was performed using the following key terms: “prurigo nodularis”, “monoclonal antibodies”, “biological treatments”, “small molecules”, and “JAK-inhibitors”.

## 3. Clinical Presentation

PN is characterized by symmetrical hyperkeratotic lesions, usually located on the extensor surfaces of the limbs and trunk [1].

The lesions manifest as crusted or excoriated, light-to-bright-red papules or nodules, with hyperpigmented margins. These lesions can range from a few millimeters to 2–3 cm in size and from just a few to hundreds in number. PN can be classified into mild (<20 lesions), moderate (20–100 lesions), and severe (>100 lesions) forms [17].

Intense pruritus lasting more than six weeks is a hallmark of the disease. Pruritus can lead to excoriations and bleeding [18]. Patients are usually unable to reach and scratch their central back; this untouched area of skin typically resembles a butterfly shape and is thus named the “butterfly sign” [17].

## 4. Conventional Therapies

Given the chronic nature of PN, long-term management is essential, and a multimodal treatment approach is often necessary [17]. Emollients are the mainstay of PN management and should always be applied regardless of disease severity. First-line treatments include topical therapies, mainly corticosteroids, calcineurin inhibitors, and phototherapy. Betamethasone valerate 0.1% has been shown to effectively reduce pruritus and flatten nodules [19]. Calcineurin inhibitors, such as pimecrolimus, are useful for long-term management, especially in sensitive areas [20]. Phototherapy, particularly PUVA and narrowband UVB, is a useful option for elderly patients with multiple comorbidities and complex medication regimens. Another approach is a modified Goeckerman regimen, which includes daily broadband UVB treatment followed by crude coal tar and topical steroids under occlusion. However, the carcinogenic potential of coal tar needs further investigation [17].

Regarding systemic therapies, treatment options include both immunomodulating and neuromodulating agents, such as immunosuppressants, gabapentinoids, antidepressants, and mu-opioid receptor antagonists [11]. Retrospective studies have demonstrated the effectiveness of methotrexate (15–20 mg weekly) and cyclosporine (2–5 mg/kg daily) in reducing pruritus and healing PN lesions, together with topical therapy [21]. Gabapentinoids, such as gabapentin and pregabalin, help alleviate pruritus by inhibiting calcium signaling, though they should be used with caution in elderly patients and those with kidney dysfunction [22]. Several studies have explored the use of opioid-receptor-modulating drugs for chronic pruritus, with mixed kappa-opioid agonist/mu-opioid antagonists nalbuphine and butorphanol both showing positive results [23]. Likewise, antidepressants like paroxetine and amitriptyline have been reported to improve pruritus, particularly in cases of severe PN with associated mental health issues [11]. As a last-resort treatment, thalidomide may be considered at doses between 50 and 150 mg daily, though its teratogenic effects and increased risk of peripheral neuropathy must be carefully considered [24].

## 5. Monoclonal Antibodies

Given the extensive number of molecules and interactions identified as playing a role in PN pathogenesis, a corresponding number of targeted therapies can be expected (Table 2). As of January 2025, dupilumab is the first FDA- and EMA-approved drug for PN. Ongoing clinical trials aim to identify new molecules that could effectively treat this condition [25].

### 5.1. Dupilumab

Dupilumab is a fully human monoclonal antibody that targets the IL-4 receptor alpha (IL-4Rα), a component of the IL-4 and IL-13 dimeric receptor [26]. By inhibiting the signaling pathways of these cytokines, dupilumab effectively dampens the Th2-driven inflammatory response, which plays a critical role in the development and persistence of PN [26].

Dupilumab has been proven effective in multiple studies. Notably, the LIBERTY-PN PRIME and PRIME 2 studies demonstrated the superiority of dupilumab as a monotherapy over a placebo in reducing pruritus as measured by the Worst Itch—Numerical Rating Scale (WI-NRS). Specifically, patients were randomized in both studies to receive either dupilumab or a placebo for 24 weeks; the primary endpoints of the studies were the percentage of patients achieving a ≥4-point reduction in the WI-NRS at 24 weeks (PRIME study) and at 12 weeks (PRIME 2 study). In the PRIME study, 60.0% of patients in the dupilumab group achieved the outcome vs. 18.4% in the placebo group, while in the PRIME 2 study, 37.2% patients reached the goal in the dupilumab group vs. 22.0% in the placebo group [27].

Enrolling a total of 311 patients, these studies also showed that dupilumab was more effective than the placebo in several secondary endpoints, such as improvement in the IGA PN-S score (Investigator’s Global Assessment for Prurigo Nodularis-Stage) at 24 weeks: in the PRIME study, 48.0% (dupilumab group) vs. 18.4% of patients (placebo group) achieved a score of 0 or 1, while in the PRIME 2 study, 44.9% vs. 15.9% of patients obtained that result. Notably, the drug exhibited an outstanding safety profile in both real-world studies and clinical trials [27,28]. In particular, in the PRIME 2 clinical trial, only conjunctivitis and herpes virus infections were common adverse effects in the dupilumab group, being reported by 3.9% and 5.2% of patients, respectively. No patients discontinued treatment because of adverse events [27].

Currently, two different observational studies are recruiting new patients to assess the maintenance of the response over time and the safety profile after a long-term time interval. The first one has a 36-month follow-up period, and it will monitor treatment discontinuation rates and their reasons (NCT05991323 [28]). The second study has a 24-month follow-up period, with the primary endpoints being a WI-NRS reduction and IGA improvement at 6 months, while the secondary endpoints evaluate the same aims at 12, 18, and 24 months (NCT06087627 [29]). Additionally, an interventional phase 3 study is enrolling patients aged 6 months to 18 years to investigate pharmacokinetics and safety in a younger population. This study includes a 24-week treatment period and a post-intervention follow-up period of 16 weeks. The main endpoints are dupilumab serum concentration (primary endpoint), incidence of adverse events (AEs), and incidence of anti-drug antibodies during the whole study duration (NCT06293053 [30]).

### 5.2. Nemolizumab

Nemolizumab is a humanized monoclonal antibody that targets the IL-31 Receptor alpha (IL-31 Rα), a part of the IL-31 dimeric receptor when paired with the oncostatin M receptor beta (OSMRβ). By doing so, it inhibits the signaling of IL-31, a cytokine that has been proven to play a role in activating inflammatory cells and directly stimulating itch-sensing neurons [31].

It is the first monoclonal antibody of its class, having been recently FDA- and EMA-approved for the treatment of PN. Nemolizumab has shown promising results in two phase 3 trials: the OLYMPIA 1 study, involving 286 patients [32], and the OLYMPIA 2 study, enrolling 274 patients [33].

Regarding the OLYMPIA 1 study, patients were randomized to receive either nemolizumab or a placebo for 24 weeks. The primary endpoints of the study were an improvement of at least 4 points from baseline in the weekly average Peak Pruritus Numerical Rating Scale (PP-NRS Scale) and an achievement of an IGA success (0/1 IGA score with a simultaneous improvement of two grades or more from baseline) at 16 weeks. The nemolizumab-treated group reported statistically significant results in both scores when compared to the placebo group: specifically, 58.4% of patients in the nemolizumab group vs. 16.7% in the placebo group achieved a PP-NRS reduction, while 26.3% vs. 7.3% of patients reported an IGA success. The side effects rate was similar in both groups, and the majority of them were mild to moderate in severity [32,33]. The most common AEs reported with a significantly higher incidence in the nemolizamun-treated group over the placebo group were headache (6.6% of patients) and AD (5.5% of patients).

Recently, another randomized controlled trial enrolling long-term responders to nemolizumab was completed (OLYMPIA DURABILITY, NCT05052983 [34]). The study included 34 patients who had previously undergone a 52-week treatment period with nemolizumab and had achieved or maintained a clinical response at week 52 (0/1 IGA score and a ≥4 improvement in the PP-NRS from baseline). Participants were randomized to receive either nemolizumab or a placebo for 24 weeks. The primary endpoint of the study was time to relapse from baseline up to 24 weeks, with relapse being defined as a ≥4 points worsening of the PP-NRS or a ≥2 points worsening of the IGA score. The study demonstrated that responsive patients at 52 weeks had lower relapse rates when continuing therapy. Supporting this, only 17% of patients maintaining nemolizumab therapy experienced a PN relapse, while 75% of patients withdrawn from treatment had a recurrence of the disease [35]. However, we will still require real-world evidence data involving a larger sample to be able to comprehensively assess the effectiveness of the treatment.

Additionally, a long-term safety trial is ongoing, with an estimated completion date in 2026. With a projected enrollment of 500 patients and a 192-week follow-up, the study will investigate the incidence of adverse events possibly occurring during nemolizumab treatment. (NCT04204616) [36].

### 5.3. Vixarelimab

Vixarelimab is a fully human monoclonal antibody that targets OSMRβ, thus inhibiting both IL-31 signaling and the oncostatin M (OSM) pathway. Oncostatin M has been identified as a mediator of cell inflammation, and levels of OSM + cells in the dermis have shown a correlation with itch severity in patients with PN [37].

In a phase 2a randomized controlled trial involving 50 patients, vixarelimab was found to significantly reduce pruritus (measured by WI-NRS) and the number of skin lesions (IGA), when compared to the placebo during an 8-week treatment course. Specifically, the primary endpoint of the study was the least squares–mean percent change from baseline (LS-PCFB) in the WI-NRS score at 8 weeks: the study reported an LS-PCFB of −50.6% in the vixarelimab group vs. −29.4% in the placebo group. The safety profile was favorable since only mild-to-moderate adverse events were reported, and they occurred in a similar frequency in the two groups; the only exceptions were upper respiratory infections, which were slightly more common in the vixarelimab group, with five patients (21.7%) experiencing these vs. two patients (7.7%) in the placebo group [37].

This study included a phase 2b sub-study, in which approximately 180 patients were randomized to receive either vixarelimab or a placebo for 16 weeks, followed by a 36-week open-label phase. The primary endpoint was the percent change from baseline in the WI-NRS at week 16, but the study also included several secondary endpoints aiming to evaluate the drug effectiveness up to 52 weeks. This portion has recently been completed, but results are not yet available (NCT03816891) [38].

### 5.4. Barzolvolimab

Barzolvolimab is a monoclonal antibody that targets KIT, a receptor found on mast cell membranes and implicated in their stimulation via Stem Cell Factor (SCF) [39]. Recently, a phase 1 study evaluated its safety and effectiveness in pruritus reduction. Patients were randomized to receive either barzolvolimab 3.0 mg/kg, barzolvolimab 1.5 mg/kg, or a placebo once, and they were then evaluated 8 weeks after drug administration; a subsequent 16-week follow-up period was established. The primary endpoints were the incidence and severity of adverse events during the study duration, while secondary endpoints were the serum concentration of the drug and mean percent change from baseline in the WI-NRS score. Barzolvolimab showed promising results in both effectiveness and safety: at week 8, 57% patients who achieved a ≥4-point reduction in the WI-NRS were in the barzolvolimab 3.0 mg/kg group, 43% in the 1.5 mg/kg group, and 25% in the placebo group; adverse events were mild to moderate in severity and were considered unrelated to treatment, except a case of anaphylactic reaction in a patient in the 3.0 mg/kg group (NCT04944862) [40].

A phase 2 study comparing barzolvolimab vs. a placebo is currently recruiting participants. Patients will be randomized to receive either barzolvolimab (two arms with different dosages) or a placebo for 24 weeks. Then, there will be a 16-week follow-up period; the primary endpoint will be an improvement of ≥4 points in the WI-NRS at week 12 (NCT06366750) [41].

### 5.5. Other Biologic Therapies

Several other biologic drugs, many of which have also been studied for the treatment of AD, are currently under investigation for PN [42].

Rocatinlimab, an OX-40 antagonist, will be evaluated in a phase 3 trial. Patients will be randomized to receive either rocatinlimab or a placebo for 52 weeks; the study will include two arms with different doses of rocatinlimab, a placebo arm, and an open-label arm. The primary endpoint will be a reduction in the WI-NRS score at 24 weeks. The study is currently in the recruiting stage (NCT06527404) [43].

SHR-1819, an IL-4Rα antagonist, will be evaluated in a phase 2/3 trial aiming to determine its efficacy and safety. Patients will be randomized into four groups receiving either the drug at three different doses or a placebo; the primary endpoints will be a ≥4-point reduction in the WI-NRS scale at 16 and 24 weeks. This study is also in the recruiting stage (NCT06554509) [44,45].

Stapokibart, another IL-4Rα antagonist, will undergo a phase 3 trial. Patients will be randomized to receive either stapokibart or a placebo, and the primary endpoint will be a ≥4-point reduction in the WI-NRS scale at 24 weeks. The study has not reached the recruiting stage yet (NCT06424470) [46,47].

MG-K10 (Comekibart), a third IL-4Rα antagonist, will also be evaluated in a phase 3 trial. The study will include a 24-week double-blind treatment period (MG-K10 or placebo), followed by a 24-week treatment phase with MG-K10 in all patients, and then an 8-week follow-up period; the primary endpoint will be a ≥4-point improvement in the WI-NRS scale at 24 weeks. This study has also not reached the recruiting stage yet (NCT06779136) [48].

Lastly, tralokinumab, an IL-13 inhibitor currently approved for the treatment of AD, has shown promising results in an open-label case series study featuring a group of 17 patients with PN-like phenotype AD. These patients were treated with tralokinumab for at least 16 weeks, and 76% (13/17) of the total reported a 0/1 IGA score at the end of the treatment period, with 47% (8/17) achieving this result within 12 weeks [49].

## 6. JAK Inhibitors

Currently, Janus kinase (JAK) inhibitors are not officially approved for PN treatment, while they have already been approved for AD treatment [50,51]. However, several promising agents, including ruxolitinib, upadacitinib, povorcitinib, abrocitinib, and tofacitinib, are undergoing clinical evaluation to assess their potential in managing PN (Table 3). These studies aim to evaluate their efficacy and safety, potentially broadening therapeutic options for PN. While ruxolitinib, upadacitinib, and abrocitinib are already approved for prurigo-like AD [52,53], their use in PN remains off-label, with available evidence limited to case reports.

### 6.1. Ruxolitinib

Ruxolitinib is a selective JAK1 and JAK2 inhibitor that disrupts the JAK-STAT pathway, reducing inflammation by modulating cytokine activity, eosinophil activity, and regulatory T-cell function [54]. Although topical ruxolitinib is already approved for non-segmental vitiligo, its therapeutic benefits for PN are being explored in multiple clinical trials.

Two ongoing phase 3, double-blind, randomized, vehicle-controlled trials (NCT05755438 [55] and NCT05764161 [56]) have completed recruitment, enrolling 190 and 204 patients, respectively. These trials focus on assessing the efficacy and safety of topical ruxolitinib in PN, evaluating the proportion of participants achieving significant clinical improvement, defined as an Investigator’s Global Assessment (IGA) score of 0 or 1 with a ≥2-point reduction and a ≥4-point decrease in Peak Pruritus Numerical Rating Scale (PP-NRS) scores.

Additionally, a phase 1 study (NCT06213831 [57]) is actively recruiting participants, specifically examining the safety, tolerability, and pharmacokinetics of ruxolitinib, with a focus on treatment-emergent adverse events (TEAEs). Another important aspect being assessed is the number of participants who experience TEAEs severe enough to require dose interruption or complete treatment discontinuation. These safety evaluations will be monitored over up to 16 weeks, including a 30-day follow-up phase.

### 6.2. Abrocitinib

Abrocitinib, an oral JAK1 inhibitor, has demonstrated efficacy in AD and is currently being investigated for PN [58]. A phase 2 open-label, nonrandomized controlled trial (NCT05038982) [59] evaluated the efficacy of abrocitinib in two groups of 10 patients each, both of which were followed until week 12. The first group consisted of patients affected by PN, while the second group consisted of patients affected by chronic pruritus of unknown origin. The primary endpoint was the percent change in weekly average PP-NRS at week 12, and secondary endpoints included the percentage of patients achieving at least a 4-point reduction in PP-NRS scores and changes in DLQI scores. The results showed a 78.3% reduction in PP-NRS scores in the PN group and the achievement of at least a 4-point reduction in PP-NRS score by 80.0% of these patients. The treatment was well tolerated, with acneiform eruption being the most common adverse event, being reported by 10% of patients in both groups [60].

### 6.3. Povorcitinib

Povorcitinib (INCB054707 [61]), an oral JAK1 inhibitor, has demonstrated potential for PN treatment. A phase 2 interventional trial (NCT05061693 [62]) enrolled 146 participants in a 16-week double-blind, placebo-controlled period followed by a 24-week single-blind extension. The primary endpoint was the percentage of patients achieving a ≥4-point reduction in the Itch-NRS score at 16 weeks. As the dose increased, the response improved: 36.1% for the drug at 15 mg, 44.4% for the drug at 45 mg, and 56.8% for the drug at 75 mg, compared to 8.1% for the placebo, showing a dose-dependent improvement in itch. The drug was well tolerated, and the most common AE, which was reported by 16.7% of patients in both active treatment groups, was headache [61,62].

Currently, povorcitinib is being investigated in two ongoing phase 3 clinical trials, STOP-PN1 (NCT06516952 [63]) and STOP-PN2 (NCT06516965 [64]), which are randomized, double-blind, placebo-controlled studies enrolling 330 participants each, aiming to evaluate its efficacy and safety in reducing pruritus and improving overall disease severity. The two studies included three treatment arms, testing two different doses of the drug compared to a placebo. The primary outcomes assessed were the proportion of participants achieving a reduction of at least 4 points in the Itch Numerical Rating Scale (Itch-NRS4) and an IGA CPG-S score of 0 or 1 with a ≥2-grade improvement from baseline (IGA-CPG-S-TS) at week 24. As of now, the results are not yet available [63,64].

### 6.4. Upadacitinib

Upadacitinib, an oral JAK1 inhibitor, is already approved for conditions such as rheumatoid arthritis, psoriatic arthritis, AD, and Crohn’s disease [65,66,67,68]. Upadacitinib has shown effectiveness in different AD phenotypes, including PN-like ones, and could also represent a valuable treatment option for patients with severe PN. It is currently being investigated for PN due to its effects on neuronal dysregulation and type 2 inflammation. A single-center, open-label study (NCT06773403 [69]) is recruiting 25 patients with moderate-to-severe PN to evaluate its efficacy over 24 weeks, focusing on pruritus severity and lesion count. The primary endpoint is the percentage of patients achieving a reduction of at least 4 points in the WI-NRS at 12 weeks in those treated with upadacitinib 15 mg orally daily, with an increase to 30 mg at week 8 in selected cases deemed necessary. As of now, the results are not yet available.

A retrospective study across 12 dermatology centers in Italy (2021–2024) assessed the efficacy and safety of upadacitinib in 21 adult patients with moderate-to-severe PN or AD with a predominant PN pattern who had previously failed other therapies. The results demonstrated a rapid and significant improvement in pruritus, along with a complete or near-complete resolution of nodules (IGA 0/1). After one month of treatment, patients experienced a mean reduction of 69.9% in the PP-NRS, with scores decreasing from 8.4 to 2.4, with the most pronounced improvement occurring in the first few days. Additionally, 86% of patients achieved a ≥4-point improvement on the PP-NRS, highlighting the strong and early efficacy of the treatment [70].

### 6.5. Tofacitinib

Tofacitinib, an oral pan-JAK inhibitor, is currently being investigated for PN treatment due to its immunomodulatory properties. A prospective, observational pilot study (NCT06201715 [71]) aims to evaluate its efficacy and safety in PN patients. This interventional trial plans to enroll 24 participants but is not yet recruiting, and its primary objectives are to evaluate the drug’s long-term effectiveness in reducing PN symptoms and to assess its safety profile in this patient population. Patients will be administered 5 mg tofacitinib tablets, to be taken orally twice daily (BID). Their response will be evaluated in a multiparametric manner, with changes at 12 weeks assessed for the following parameters: IGA, PAS (Prurigo Activity Score), VAS (Visual Analogue Scale), NRS, VRS (Verbal Rating Scale), DLQI, and Itchy QoL (Itchy-Specific Quality of Life). Several published case reports describe patients with PN treated with tofacitinib, demonstrating improvements in skin lesions and significant reductions in NRS and DLQI scores during treatment [72,73,74].

## 7. Conclusions

The treatment of PN remains challenging, due to the limited therapeutic options. Currently, dupilumab and nemolizumab are the only FDA- and EMA-approved monoclonal antibodies for severe PN. Vixarelimab and JAK inhibitors have shown promising efficacy data in clinical trials and real-world case series. However, these results should be confirmed by larger and longer observations. Also, currently, no innovative treatments are available for the treatment of adolescents and children. The development of clinical trials to evaluate the role of biologics and small molecules in these populations is strongly required.

## Figures and Tables

**Table 1:** Conditions associated with prurigo nodularis.

Category	Associated Conditions
Dermatological	Atopic dermatitis Contact dermatitis Eczema
Systemic	Diabetes mellitus, Chronic kidney disease Liver diseases including hepatitis C Thyroid disorders Untreated HIV infection Lymphomas (Hodgkin and non-Hodgkin); certain cancers including skin malignancies
Neurological	Peripheral neuropathy Multiple sclerosis
Psychiatric	Anxiety disorders Depression Obsessive–compulsive disorder
Other	Vitamin deficiencies Parasitic infestations Bacterial infections (e.g., tuberculosis) Drug reactions including some chemotherapy agents

**Table 2:** List of completed and ongoing trials, categorized by biologic drug of interest.

Candidate Drug	Trial Identifier	Trial Phase, Status	Arms and Intervetions	Primary Endpoint	Outcome
Dupilumab	NCT04183335 LIBERTY PN-PRIME	3, completed	Drug, 300 mg subcutaneous injection once every 2 weeks: n = 75 Placebo: n = 76	Percentage of patients achieving a WI-NRS reduction of ≥4 points at 24 weeks	Drug 300 mg once every 2 weeks: 60.0% Placebo: 18.4%
	NCT04202679 PRIME2	3, completed	Drug, 300 mg subcutaneous injection once every 2 weeks: n = 78 Placebo: n = 82	Percentage of patients achieving a WI-NRS reduction of ≥4 points at 12 weeks	Drug 300 mg once every 2 weeks: 37.2% Placebo: 22.0%
	NCT05991323 GLOBOSPIN	Observational study, recruiting	Drug, subcutaneous injection: n = 300 (estimated)	(1) Reasons for treatment iniations; (2) frequency of treatment modifications; (3) patients discontinuing treatment; (4) reasons behind discontinuations; (5) patients hospitalized due to PN. (All outcomes measured from baseline up to 36 months)	
	NCT06087627 CLEAR PN	Observational study, recruiting	Drug, subcutaneous injection: n = 150 (estimated)	(1) Percentage of participants with an IGA score of 0 or 1 at 6 months. (2) Percentage of participants achieving a ≥4 points improvement in the WI-NRS from baseline at 6 months	
	NCT06293053	3, recruiting (patients aged 6 months to 18 years)	Drug, subcutaneous injection: n = 18 (estimated)	Concentration of dupilumab in serum from day 1 to week 40	
Nemolizumab	NCT04501666 OLYMPIA 1	3, completed	Drug 30 mg (2 subcutaneous injections at w0, then 1 injection every 4 weeks in patients weighing < 90 kg or 2 injections every 4 weeks in patients weighing ≥ 90 kg): n = 190 Placebo: n = 96	(1) Number of participants with an improvement of ≥4 points from baseline in PP-NRS at 16 weeks; (2) number of participants achieving an IGA success at 16 weeks	(1) Drug: 58.4%, Placebo: 16.7%; (2) Drug: 26.3%, Placebo: 7.3%.
	NCT04501679 OLYMPIA 2	3, completed	Drug 30 mg (2 subcutaneous injections at w0, then 1 injection every 4 weeks in patients weighing < 90 kg or 2 injections every 4 weeks in patients weighing ≥ 90 kg): n = 183 Placebo: n = 91	(1) Number of participants with an improvement of ≥4 points from Baseline in PP-NRS at week 16; (2) Number of participants achieving an IGA success at week 16	(1) Drug: 56.3%, Placebo: 20.9%; (2) Drug: 37.7%, Placebo: 11.0%.
	NCT05052983 OLYMPIA DURABILITY	3, completed (study in long-term responders)	Drug 30 mg (1 subcutaneous injection every 4 weeks in patients weighing < 90 kg, 2 injections every 4 weeks in patients weighing ≥ 90 kg): n = NA Placebo: n = NA	Time from baseline to relapse meeting at least 1 of the defined criteria (from baseline up to 24 weeks)	Drug: 17% Placebo: 75%
	NCT04204616	3, active, not recruiting	Drug 30 mg (1 subcutaneous injection every 4 weeks in patients weighing < 90 kg, 2 injections every 4 weeks in patients weighing ≥ 90 kg): n = 500 (estimated)	Incidence of adverse events (AEs) by severity from baseline up to 192 weeks	Results not yet available
Vixarelimab	NCT03816891	2a, completed	Drug 360 mg, subcutaneous injections (720 mg loading dose at w0, then 360 mg once a week): n = 23 Placebo: n = 26	Percent change from baseline in WI-NRS at week 8 (Least-squares (LS); mean percent change from baseline (PCFB))	Drug: −50.6% Placebo: −29.4%
	NCT03816891	2b, completed	(A) Drug 540 mg, subcutaneous injections, once every 4 weeks during the double-blind period (DBP); (B) Drug 360 mg, subcutaneous injections, once every 4 weeks during the DBP; (C) Drug 120 mg, subcutaneous injections, once every 4 weeks during the DBP; (D) Placebo; (E) Drug 360 mg, subcutaneous injections, once every 2 weeks during the open-label extension. (Numbers NA)	Percent change from baseline in WI-NRS at week 16	Results not yet available
Barzolvolimab	NCT04944862	1b, completed	(A) Drug, 3.0 mg/kg, single intravenous injection at w0: n = 9; (B) Drug, 1.5 mg/kg, single intravenous injection at w0: n = 7; (C) Placebo: n = 8.	Proportion of participants with improvement of ≥4 points from baseline in WI-NRS at week 12	(1) Drug 3.0 mg/kg: 57%; (2) Drug 1.5 mg/kg group: 43%; (3) Placebo: 25%.
	NCT06366750	2, recruiting	(A) Drug, subcutaneous injections (450 mg loading dose at w0, then 150 mg every 4 weeks); (B) Drug, subcutaneous injections (450 mg loading dose at w0, then 300 mg every 4 weeks); (C) Placebo.	Safety and tolerability as assessed by the incidence and severity of adverse events from day 1 to day 169	One case of anaphylactic reaction in the 3.0 mg/kg group; generally, mild-to-moderate adverse events mostly consistent with PN comorbidities in all groups
Rocatinlimab	NCT06527404	3, recruiting	(A) Drug, subcutaneous injection, dose 1 during treatment period A and treatment period B (blinded treatment); (B) Drug, subcutaneous injection, dose 2 during treatment period A and treatment period B (blinded treatment); (C) Placebo during treatment period A and treatment period B (blinded treatment); (D) Drug, subcutaneous injection, dose 1 during treatment period B (open-label treatment).	Number of participants achieving a reduction from baseline in the weekly average Daily Itch Score at week 24	
SHR-1819	NCT06554509	2/3, recruiting	(A) Drug, injection dose A; (B) Drug, injection dose B; (C) Drug, injection dose C; (D) Placebo.	(1) Proportion of subjects with a ≥4-point reduction from baseline in WI-NRS at week 16 (phase 2); (2) Proportion of subjects with a ≥4-point reduction from baseline in WI-NRS at week 24 (phase 3).	
Stapokibart	NCT06424470	3, not yet recruiting	Drug, subcutaneous injection; Placebo	Propotion of subjects with improvement of ≥4 points from baseline on WI-NRS at week 24	
MG-K10 (Comekibart)	NCT06779136	3, not yet recruiting	Drug, subcutaneous injection every 4 weeks; placebo (after week 24 all patients switch to drug injection)	Proportions of subjects achieving a WI-NRS improvement of ≥4-point from baseline at week 24	

**Table 3:** List of completed and ongoing trials, categorized by JAK inhibitors of interest.

Candidate Drug	Trial Identifier	Trial Phase, Status	Arms and Intervetions	Primary Endpoint	Outcome
Ruxolitinib	NCT05755438	3, active not recruiting	Drug 1.5% cream: n = 102 Placebo: n = 102	Percentage of patients with WI-NRS reduction of ≥4 points at 12 weeks	Results not yet available
	NCT05764161	3, active not recruiting	Drug 1.5% cream: n = 95 Placebo: n = 95	Percentage of patients with WI-NRS reduction of ≥4 points at 12 weeks	Results not yet available
	NCT06213831	1, recruiting	Drug 1.5% cream: n = 24	Percentage of patients with TEAEs over 16 weeks	
Abrocitinib	NCT05038982	2, completed	Drug 200 mg oral daily: n = 10	Percentage reduction in weekly average PP-NRS at 12 weeks	Drug 200 mg oral daily: 78.3%
Povorcitinib	NCT05061693	2, completed	(A) Drug 15 mg oral daily: n = 36; (B) Drug 45 mg oral daily: n = 35; (C) Drug 75 mg oral daily: n = 37; (D) Placebo: n = 37.	Percentage of patients with Itch-NRS4 reduction of ≥4 points at 16 weeks	Drug 15 mg oral daily: 36.1% Drug 45 mg oral daily: 44.4% Drug 75 mg oral daily: 56.8% Placebo: 8.1%
	NCT06516952 STOP-PN1	3, recruiting	(A) Drug Dose 1: n = 110; (B) Drug Dose 2: n = 110; (C) Placebo: n = 110.	Percentage of patients achieving Itch-NRS4 and IGA-CPG-S-TS at week 24	
	NCT06516965 STOP-PN2	3, recruiting	(A) Drug Dose 1: n = 110; (B) Drug Dose 2: n = 110; (C) Placebo: n = 110.	Percentage of patients achieving Itch-NRS4 and IGA-CPG-S-TS at week 24	
Upadacitinib	NCT06773403	4, recruiting	Drug 15mg oral daily, optional increase to 30mg at week 8: n = 25	Percentage of patients with WI-NRS reduction of ≥4 points at 12 weeks	
Tofacitinib	NCT06201715	Not applicable, not yet recruiting	Drug 5 mg oral BID: n = 24	Percentage of patients with IGA, PAS, VAS, NRS, VRS, DLQI, and Itchy QoL change at 12 weeks	

## Data Availability

No new data were created or analyzed in this study. Data sharing is not applicable to this article.
